# Modulation of Type-I Interferon Response by hsa-miR-374b-5p During Japanese Encephalitis Virus Infection in Human Microglial Cells

**DOI:** 10.3389/fcimb.2019.00291

**Published:** 2019-08-09

**Authors:** Meghana Rastogi, Sunit K. Singh

**Affiliations:** Molecular Biology Unit, Institute of Medical Sciences, Banaras Hindu University, Varanasi, India

**Keywords:** JEV, human microglial cells, hsa-miR-374b-5p, type-I interferon response, viral immune evasion

## Abstract

Japanese Encephalitis virus (JEV) is a neurotropic ssRNA virus, belonging to the *Flaviviridae* family. JEV is one of the leading causes of the viral encephalitis in Southeast-Asian countries. JEV primarily infects neurons however, the microglial activation has been reported to further enhance the neuroinflammation and promote neuronal death. The PI3K/AKT pathway has been reported to play an important role in type-I interferon response via IRF3. Phosphatase and tensin homolog (PTEN), a negative regulator of PI3K/AKT pathway, participates in microglial polarization and neuroinflammation. The microRNAs are small non-coding endogenously expressed RNAs, which regulate the gene expression by binding at 3′ UTR of target gene. The human microglial cells were infected with JEV (JaOArS982 strain) and up-regulation of microRNA; hsa-miR-374b-5p was confirmed by qRT-PCR. The genes in PI3K/AKT pathway, over-expression and knock-down studies of hsa-miR-374b-5p with and without JEV infection were analyzed through immuno blotting. The regulatory role of hsa-miR-374b-5p on the expression of type-I interferon was determined by luciferase assays. JEV infection modulated the expression of hsa-miR-374b-5p and PI3K/AKT pathway via PTEN. The over-expression of hsa-miR-374b-5p suppressed the PTEN while up-regulated the AKT and IRF3 proteins, whereas, the knockdown rescued the PTEN expression and suppressed the AKT and IRF3 proteins. The modulation of hsa-miR-374b-5p regulated the type-I interferon response during JEV infection. In present study, we have shown the modulation of PTEN by hsa-miR-374b-5p, which regulated the PI3K/AKT/IRF3 axis in JEV infected microglial cells.

## Introduction

The Japanese Encephalitis virus (JEV) is a mosquito-borne Flavivirus belongs to *Flaviviridae* family. The *Flaviviridae* family also includes Dengue virus (DENV), West Nile Virus (WNV), Yellow Fever Virus (YFV), Tick Borne Encephalitis Virus (TBEV), and ZIKA Virus (ZIKV) (Gould and Solomon, 2008). JEV is one of the leading causes of viral encephalitis in Southeast-Asian countries, with an annual incidence of 70,000 and case fatality rate of 30–50%, predominantly affecting children and elderly people (Campbell et al., 2011). JEV has devised several molecular strategies to evade the hosts immune response in order to establish itself successfully inside the host (Aleyas et al., 2010; Yang et al., 2011).

Microglial cells are the resident macrophages in the central nervous system (CNS) and play roles in phagocytosis, immune surveillance, and antigen presentation (Colonna and Butovsky, 2017; Salter and Stevens, 2017). JEV has been reported to persist in microglial cells, which could be a plausible reservoir for infection (Thongtan et al., 2010). The JEV primarily infects and kills neurons and further enhances the neuroinflammatory events by producing chemokines and cytokines, which in turn activates microglial cells and leads to neuronal death in a bystander fashion (Ghoshal et al., 2007; Chen et al., 2010, 2012a). The microglial activation is regarded as one of the signs of neuroinflammation during viral infections (Conrady et al., 2013; Drokhlyansky et al., 2017; Mathur et al., 2017; Dello Russo et al., 2018). MicroRNAs are the endogenously expressed small non-coding RNAs, which regulate the gene expression by binding to the 3′ UTR of target mRNA (Ambros, 2004; Singh et al., 2008). We and others have previously reported the perturbation in cellular microRNA expression patterns during JEV infection for the purpose of replication and immune evasion (Cullen, 2013; Sharma et al., 2015, 2016; Rastogi et al., 2018).

The viral infection to microglial cells activates multiple signaling cascades, where the first-line-of-defense, type-I interferon response, plays a major role in orchestrating the anti-viral signaling by producing the IFN-α/β (Samuel, 2001; Randall and Goodbourn, 2008). The binding of these pleiotropic cytokines to their receptor IFNAR (IFNαR1/R2) stimulates the ISGs (IFN-stimulated genes) by binding to the IFN-stimulated response *cis*-elements (ISREs) inside the nucleus (Aaronson and Horvath, 2002). The type-I interferon responses have been reported to be transcriptionally regulated by RIG-I/Mda5 pathway, TLR3 and TLR4 mediated TRIF (Jiang et al., 2014) and TLR7/8 and TLR9 pathways. TRIF pathway leads to the activation of interferon regulatory factor 3 (IRF3), whereas the TLR7/8 and TLR9 pathway leads to the activation of interferon regulatory factor 7 (IRF7) (Daffis et al., 2008). We and others have reported the type I interferon promotes neuroinflammation in activated microglial cells (Furr and Marriot, 2012; Main et al., 2016) as well as in the JEV infected microglial cells (Manocha et al., 2014; Sharma et al., 2015; Lannes et al., 2017).

The involvement of the PI3K/AKT signaling has been reported in various cellular functions; however its dysregulation has been reported in neuroinflammation (Tang et al., 2017; Yang et al., 2017). In addition, the AKT/IRF3 axis has been studied in Traumatic Brain Injury (TBI) (Wang et al., 2015) and auto-inflammatory disorders (Oh et al., 2018). The activation of PI3K/AKT pathway during viral infection has been correlated to viral replication, viral entry (Esfandiarei et al., 2004; Lee et al., 2005) and virus-induced apoptosis (Esfandiarei et al., 2004; Lee et al., 2005; Schabbauer et al., 2008). In addition, the activation of IRF3 via PI3K/AKT pathway has been reported in the production of IFN-β response (Joung et al., 2011; Lu et al., 2011; Tarassishin et al., 2011a; Cianciulli et al., 2016; Wang et al., 2017).

The PTEN (phosphatase and tensin homolog) is a tumor suppressor protein and a negative regulator for PI3K/AKT pathway, where the phosphorylation of PTEN negatively regulates PI3K/AKT pathway (Vazquez et al., 2000, 2001). The PTEN protein has been reported for its role in neuropathic pain, neuroinflammation, and modulating the microglial polarization through PTEN/AKT axis (Zhao et al., 2014; Huang et al., 2015; Wang et al., 2015; Cao et al., 2017). Although, PTEN has been studied in various tumors (Cheng et al., 2015; Yu et al., 2016), oncolytic viruses (Wu et al., 2017) but its role in microbial innate immunity has been recently identified, where it induces the interferon responses. The type-I interferon inducing capability of PTEN rely on its phosphatase activity, where the phosphorylation of PTEN activates the type-I interferon response (Li et al., 2016). The microRNA, hsa-miR-374b-5p has been identified in various cancers, neurodegeneration like Alzheimer's, hypoxic-ischemic encephalopathy and epilepsy etc. (Bian et al., 2019).

This is the first report where the role of PTEN has been highlighted in the modulation of type-I interferon response during JEV infection. We demonstrated the suppression of PTEN by microRNA, hsa-miR-374b-5p at 24 h of JEV infection in human microglial cells. The suppression of a negative regulator induced the PI3K/AKT pathway and promoted the type-I anti-viral response via IRF3. The suppression of type-I anti-viral response during later stages of the infection process might be the strategy of JEV to subvert the anti-viral response.

## Materials and Methods

### Cell Culture

The human microglial cells (Dello Russo et al., 2018), PS (porcine kidney cells) and Vero cells were cultured in DMEM (GIBCO) supplemented with heat inactivated 10% FBS (GIBCO) and 100 U/ml of penicillin, 100 mg/ml streptomycin and 29.2 mg/ML. L-Glutamine (GIBCO) in humidified CO_2_ incubator at 37°C. The human microglial cell line was the kind gift from Prof. Anirban Basu, National Brain Research Centre (NBRC), Manesar, Haryana.

### The Virus Propagation, Titration, and Infection

The JaOArS982 strain of JEV was propagated in suckling BALB/c mice at NBRC, Manesar. The *in-vitro* propagation was done in the Vero cells at the MOI of 0.1 in the incomplete DMEM medium. The incomplete DMEM cell culture media was replaced by complete DMEM post infection (2 h) and left in CO_2_ incubator for 5 days or until 80% cell death was observed. The virus were titrated in PS cells by using plaque assay as described elsewhere (Sharma et al., 2015).

All of the JEV infection experiments were conducted in human microglial cells at the MOI of 5 in 6 well cell culture plates at the cell density of 0.3 × 10^6^ cells/well in incomplete DMEM for 2 h. The incomplete DMEM was changed to complete DMEM and the cells were harvested at 12, 24, and 48 h post JEV infection and stored at −80°C until further use.

### RNA Isolation, Micro RNA Expression and Real-Time PCR

The Qiagen miRNeasy kit (#217004; Qiagen, Venlo, Netherlands) was used for the isolation of total RNA from the microglial cells harvested at different time points. The complementary DNA (cDNA) was prepared by using Superscript II reverse transcriptase system (#11904-018, Invitrogen, CS, USA) using the manufacturer's protocol. The thermal cycles for synthesizing cDNA were: 65°C-5 min, 25°C-10 min, 42°C-50 min, and 70°C-10 min, then, RNase H treatment-20 min at 37°C. The JEV infection in the human microglial cells was confirmed by q-PCR against the JEV NS3 gene, normalized to GAPDH by using Agilent Brilliant III ultrafast SYBR green master mix (#600882, Agilent Technologies, California, US) (Table 1).

**Table 1:** List of primers, microRNA oligos, and scramble sequence.

**Viral NS3 Forward**	**5′ AGAGCGGGGAAAAAGGTCAT 3′**
Viral NS3 Reverse	5′ TTTCACGCTCTTTCTACAGT 3′
GAPDH Forward	5′ ATGGGGGAAGGTGAAGGTCG 3′
GAPDH Reverse	5′ GGGGTCATTGATGGCAACAATA 3′
Mimics of hsa-miR-374b-5p	5′ AAAUGGCAUUAUAUAUAUUAUA 3′
Scramble of hsa-miR-374b-5p	5′ CAGAUUCUAUUUGCCCAAGAA 3′

To study the microRNA expression, the cDNA was synthesized by using MultiScribe TaqMan Reverse Transcriptase (#4366596; Applied Biosystems, Waltham, MA, USA) along with hsa-miR-374b-5p specific primers according to manufacturer's protocol. The microRNA expression was analyzed using a real time PCR machine (Agilent AriaMx) by using a hsa-miR-374b-5p-specific TaqMan probe and universal PCR master mix (#4324018; Applied Biosystems). The expression of hsa-miR-374b-5p was normalized by endogenous control RNU6b expression.

### Protein Isolation, Estimation, and Immuno-Blotting

The microglial cells harvested at different time points were used for protein isolation by using complete RIPA buffer (#89900 Pierce, Thermo Fischer Scientific) and protease inhibitor cocktail (#80650123 GE Biosciences). Briefly, the pellets were dissolved in RIPA buffer, sonicated for 5 min and centrifuged at 10,000 × g for 20 min at 4°C. The supernatant was collected, and the protein was estimated by BCA assay (#23227 Pierce, Thermo Fischer Scientific). The protein was run on 10% SDS-PAGE and then transferred to PVDF membrane (#IPVH00010 Immobilon, Merck, Millipore). The blocking was done in 5% skimmed milk (#sc-2324 Santa Cruz) for an hour and the specific primary antibody was incubated in 5% skimmed milk at 4°C overnight. The anti-PTEN antibody (# 9559S CST), anti-AKT antibody (#2920S CST), anti-p-AKT antibody (#9271), anti-IRF3 antibody (#4302S CST), and anti-β-tubulin antibody (# 250904 ABBiotec) were given in a 1:1000 dilution while anti-p-PTEN (#9549P CST) and anti-p-IRF3 (#4947S CST) were blocked and incubated in 5% BSA, in 1:1000 dilutions overnight. The goat-anti-rabbit and mouse anti-goat secondary antibody were given at 1:50,000 for 2–3 h at room temperature in 5% skimmed milk and 5% BSA. The blots were developed in ChemiDoc (Azure Biosystems) by using west femto ECL substrate (#34095 Super Signal West Femto Thermo Fischer Scientific) at different exposures. The Image J (ver: 1.42q) software has been used for densitometry of immunoblots.

### Over-expression and Inhibition Studies

The human microglial cells were seeded in 6 well plate at the density of 0.4 × 10^6^ cells/well, one day prior to transfection. The cells were transfected with 200 pmol of hsa-miR-374-5p mimics (Bioserve, Hyderabad, India) and 200 pmol of hsa-miR-374b-5p scramble (ILS) at the confluence of 70% by using chemical method, Lipofectamine 2000 (#11668-019; Invitrogen, Carlsbad, CA, USA). The scramble sequence and mock transfected cells were used as controls. The overexpression study was confirmed by quantitative real-time PCR using TaqMan microRNA assay after 48 h post-transfection. The gene targets were confirmed by Immuno-blotting. The microRNA Inhibitor studies were performed by transfecting the anti-miR against (200 pmol) hsa-miR-374b-5p (#AM11339, Invitrogen, Carlsbad, CS, USA) and anti-miR scramble cy3 negative control (#AM17011, Invitrogen, Carlsbad, CS, USA) by using Lipofectamine 2000 (#11668-019; Invitrogen, Carlsbad, CA, USA) and cells were harvested 48 h post transfection. The inhibitor studies were confirmed by quantitative real-time PCR using TaqMan microRNA assay. The gene targets were confirmed by Immuno-blotting. The transfected cells were infected post 24 h at the MOI of 5 for another 24 h. The cells were harvested 48 h post-transfection.

### Luciferase Assay

The human microglial cells were seeded in 6 well plates at the density of 0.4 × 10^6^ cells/well and were co-transfected with IFN-β-luciferase plasmid (1 μg) and normalized by β-gal (700 ng) vector. For over-expression and inhibitor studies, 200 pmol of hsa-miR-374b-5p mimics, scramble, anti-miR and cy3 negative control were co-transfected in human microglial cells and the luciferase activity was measured post 48 h. The cells were infected after 24 h post transfection at MOI 5.

The luminescence was measured by using a Luciferase assay kit (#E4030; Promega, Madison, WI, USA) as per the manufacturer's protocol and the luminescence activity was measured on multimode reader (Synergy HTX, Bio-Tek). The luminescence values were normalized by using β-gal plasmid vector. The β-galactosidase activity was measured at the absorbance of 420 nm by plate reader (imark plate reader, Bio-Rad).

### Statistical Analysis

All experiments were conducted in triplicate (*n* = 3) and one-tailed, paired Student's *t*-test was used to make comparison between data sets. The data are shown as mean ± S.E from three independent experiments and data was considered significant when *P* < 0.05; ^*^ denotes *P* < 0.05, ^**^ denotes *P* < 0.01, ^***^ denotes *P* < 0.001.

## Results

### JEV Infection Modulates the PI3K/AKT Pathway in Human Microglial Cells

**Figure 1:**
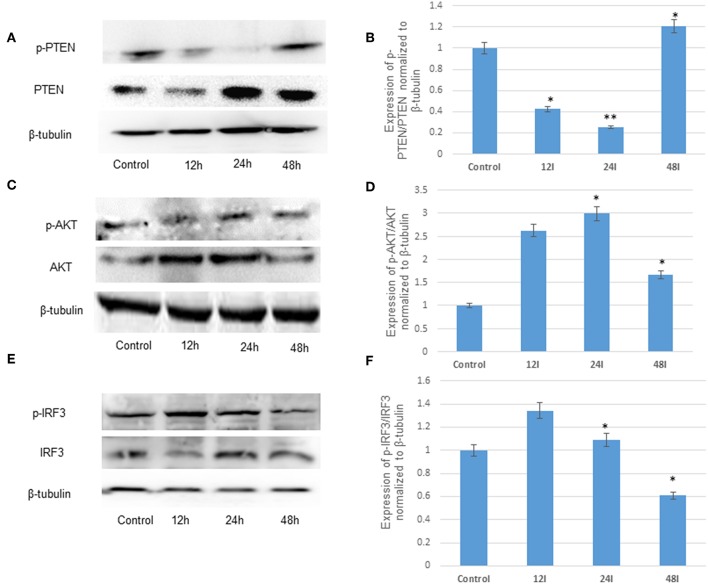
JEV infection suppresses the negative regulator, PTEN and modulates PI3K/AKT pathway in human microglial cells. **(A)** Immunoblot of p-PTEN/PTEN at 12, 24, and 48 h post JEV infection compared to control **(B)** densitometry. **(C)** Immunoblot of p-AKT/AKT 12, 24, and 48 h post JEV infection compared to control **(D)** densitometry. **(E)** Immunoblot of p-IRF3/IRF3 12, 24, and 48 h post JEV infection compared to control **(F)** densitometry. All experiments were performed in triplicate (*n* = *3*). The data are shown as mean ± S.E from three independent experiments. The fold change is significant where * denotes *P* < 0.05, ** denotes *P* < 0.01, *** denotes *P* < 0.001.

We and others have previously reported the JEV infection in the brain resident macrophages (microglial cells) (Thongtan et al., 2010; Manocha et al., 2014; Sharma et al., 2015; Lannes et al., 2017). The modulation of several cell signaling pathways leading to various neuroinflammatory events during JEV infection has been reported (Manocha et al., 2014; Sharma et al., 2015). The recent reports have highlighted the induction of immune responses through PI3K/AKT pathways (Sarkar et al., 2004; Hazeki et al., 2007; Polumuri et al., 2007; Radler et al., 2017) involving the AKT and IRF3 genes (Tarassishin et al., 2011b). PTEN is a negative regulator of PI3K/AKT pathway, and we observed the suppression of p-PTEN/PTEN at 12 and 24 h (60 and 70% respectively) post JEV infection, whereas the expression of PTEN increased at 48 h compared to un-infected human microglial cells (Figures 1A,B). In addition, we demonstrated the modulation of AKT and IRF3 proteins at different time points by immunoblotting (Figures 1C–F). At early time point, 12 and 24 h post JEV infection, the p-AKT/AKT and p-IRF3/IRF3 proteins were shown to be up-regulated, whereas at later stages of the infection progress (at 48 h), the expression of p-AKT/AKT and p-IRF3/IRF3 have shown the decreasing trend (Figures 1C–F). Therefore, the JEV infection in human microglial cells modulates the PI3K/AKT pathway via PTEN during early and late courses of infection.

### JEV Infection in Human Microglial Cells Modulates the Micro RNA-374b-5p Expression

**Figure 2:**
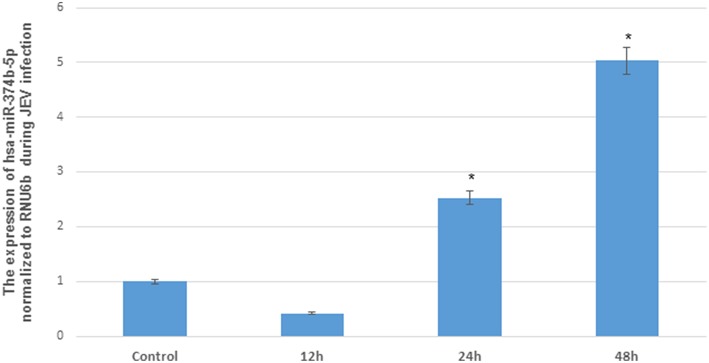
The differential expression of hsa-miR-374b-5p after JEV (JaOARs982 strain) infection in human microglial cells. The change in hsa-miR-374b-5p after JEV (JaOARs982 strain) infection at different time points. The qRT-PCR by using TaqMan microRNA assay shows the up-regulation from 24 h to 48 h by 2.5–5-folds compared to control. The data are shown as mean ± S.E from three independent experiments (*n* = *3*). The fold change is significant where * denotes *P* < 0.05, ** denotes *P* < 0.01, *** denotes *P* < 0.001.

The microRNAs have been reported to modulate the interferon response during viral infections (Buggele et al., 2013). In addition, hsa-miR-374b-5p has been reported to increase PTEN expression in various types of cancers (Li et al., 2015; Long et al., 2018). In our results, we demonstrated the gradual increase of microRNA hsa-miR-374b-5p expression levels from 24 to 48 h post JEV infection with the fold change of 2.5 to 5 (Figure 2). The up-regulation of microRNA hsa-miR-374b-5p at 24 h and suppression of PTEN at 24 h during JEV infection may be co-related (Figures 1A,B).

### The hsa-miR-374b-5p Targets PTEN and Modulates the Expression of AKT and IRF3

**Figure 3:**
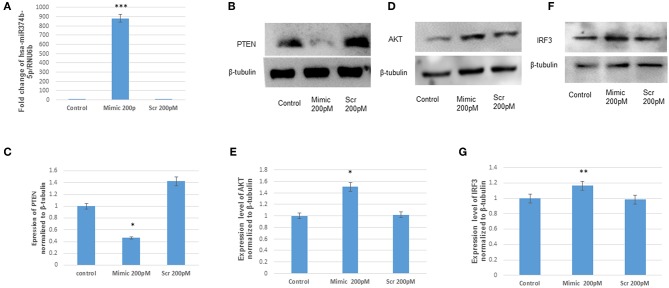
The hsa-miR-374b-5p targets PTEN and activates the expression of AKT and IRF3. **(A)** qRT-PCR of hsa-miR-374b-5p overexpressed in human microglial cells by using RNA oligos at 200 pmol conc. **(B)** The immunoblots showing the suppression of PTEN by 70%, 48 h post transfection. **(C)** The densitometry of PTEN. **(D)** The immunoblots showing up-regulation of AKT by 1.5-fold. **(E)** Densitometry of AKT. **(F)** The immunoblots showing up-regulation of IRF3 by 1.2-fold upon overexpression **(G)** densitometry of IRF3. The data are shown as mean ± S.E from three independent experiments (*n* = *3*). The fold change is significant where * denotes *P* < 0.05, ** denotes *P* < 0.01, *** denotes *P* < 0.001.

To confirm the targeting of microRNA with PTEN, the hsa-miR-374b-5p mimics and scrambled (hsa-miR-374b-5p) were transfected in human microglial cells and incubated for 48 h (Table 1). The up-regulation of mimics was confirmed by TaqMan microRNA assay, while no changes were observed in the samples transfected with scrambled sequences (Figure 3A). The expression of PTEN protein was reduced by 70% (Figures 3B,C), whereas the other proteins, AKT and IRF3 were up regulated by 1.5 and 1.2-fold post transfection (Figures 3D–G). The microRNA, hsa-miR-374b-5p targeted to PTEN and modulated the genes involve in activation of interferon response via PI3K/AKT pathway.

### The anti-miR-374b-5p Rescues the Expression of PTEN and Suppresses the Expression of AKT and IRF3

**Figure 4:**
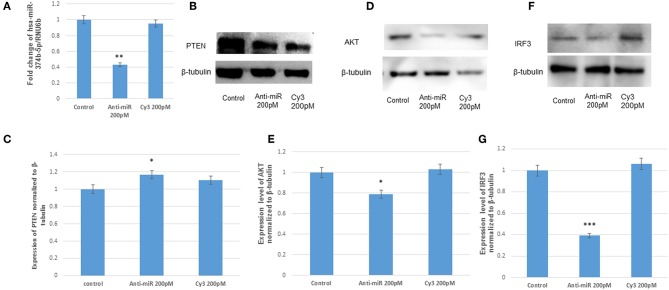
The anti-miR-374b-5p rescues the expression of PTEN and suppresses the expression of AKT and IRF3. The knock down studies of hsa-miR-374b-5p **(A)** qRT-PCR knockdown of miR-374b-5p in human microglial cells by using inhibitors at 200 pmol conc. **(B)** The immuno blots showing the rescue of PTEN expression 48 h post transfection. **(C)** The densitometry of PTEN. **(D)** The immuno blots shows the suppression of AKT by 20% during transfection. **(E)** Densitometry of AKT. **(F)** The immuno blots shows the suppression of IRF3 by 60% during transfection. **(G)** Densitometry of IRF3. The data are shown as mean ± S.E from three independent experiments (*n* = *3*). The fold change is significant where * denotes *P* < 0.05, ** denotes *P* < 0.01, *** denotes *P* < 0.001.

To further validate the targeting of microRNA, hsa-miR-374b-5p on its target PTEN, the hsa-miR-374b-5p was silenced by using microRNA inhibitors (200 pM) and cy3 anti-miR negative control (200 pM) in human microglial cells for 48 h utilizing transfection approaches. The suppression of microRNA was confirmed by TaqMan microRNA assay with around 60% suppression, while no changes were observed in the cy3 anti-miR control (Figure 4A). The immunoblot analysis during the anti-miR treatment rescued the expression of PTEN by 1.1-fold (Figures 4B,C) and the expressions of AKT and IRF3 were suppressed by 20 and 60% post transfection (Figures 4D–G). Therefore, we concluded the hsa-miR-374b-5p alone can specifically modulate the expression of PI3K/AKT pathway.

### The Effect of hsa-miR-374b-5p Over-expression and Knockdown During JEV Infection

**Figure 5:**
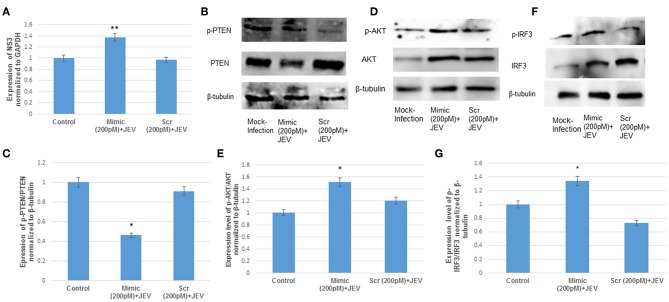
The over-expression of hsa-miR-374b-5p during JEV infection. **(A)** qRT-PCR to confirm the JEV infection post transfection by quantifying the JEV NS3 levels. **(B)** Expression levels of p-PTEN/PTEN suppress by 50% during over-expression of microRNA mimics post JEV infection. **(C)** Densitometry of p-PTEN/PTEN. **(D)** The p-AKT/AKT expression up-regulates by 1.4-folds during over-expressing the microRNA mimics and JEV infection. **(E)** Densitometry plot of p-AKT/AKT. **(F)** The p-IRF3/IRF3 expression up-regulates up to 1.3-fold during over-expressing the microRNA mimics and JEV infection. **(G)** Densitometry plot of p-IRF3/IRF3. The data are shown as mean ± S.E from three independent experiments (*n* = *3*). The fold change is significant where * denotes *P* < 0.05, ** denotes *P* < 0.01, *** denotes *P* < 0.001.

**Figure 6:**
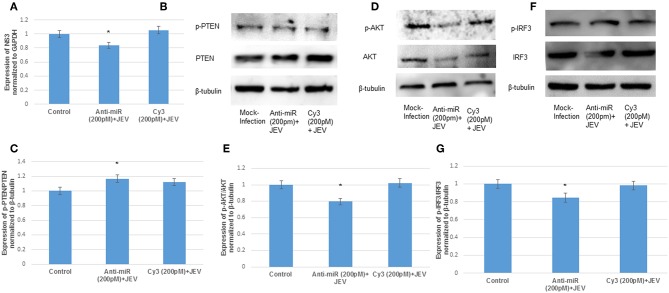
The knockdown of hsa-miR-374b-5p during JEV infection. **(A)** The qRT-PCR to confirm the JEV infection post transfection. **(B)** Expression levels of p-PTEN/PTEN rescues up to 1.2-fold during anti-miR transfection post JEV infection. **(C)** The densitometry plot of p-PTEN/PTEN. **(D)** The p-AKT/AKT expression suppresses by 20% during anti-miR transfection post JEV infection. **(E)** The densitometry plot of p-AKT/AKT. **(F)** The p-IRF3/IRF3 expression suppresses by 20% during knockdown of hsa-miR-374b-5p post JEV infection. **(G)** Densitometry plot of p-IRF3/IRF3 proteins. The data are shown as mean ± S.E from three independent experiments (*n* = *3*). The fold change is significant where * denotes *P* < 0.05, ** denotes *P* < 0.01, *** denotes *P* < 0.001.

The hsa-miR-374b-5p was overexpressed and knocked down in the microglial cells during the JEV infection to illustrate the role of microRNA. Since, the hsa-miR-374b-5p expression increased at 24 h, the JEV infection was given 24 h post transfection at MOI 5 in human microglial cells. The infection was confirmed by NS3 q-PCR post 48 h of transfection (Figures 5A, 6A). The viral replication, as measured by the expression levels of NS3, increased during the over-expression of microRNA mimics, while it suppressed by inhibitors (Figures 5A, 6A). The effect of the hsa-miR-374b-5p mimics suppressed the p-PTEN/PTEN levels (Figures 5B,C) and up-regulated the p-AKT/AKT and p-IRF3/IRF3 levels (Figures 5D–G), whereas as a result of the suppression by anti-miR inhibitors, the expression levels were rescued for p-PTEN/PTEN (Figures 6B,C) and suppressed the expression of p-AKT/AKT and p-IRF3/IRF3 levels (Figures 6D–G).

### The Activation of type-I Interferon Response During JEV Infection in Human Microglial Cells

**Figure 7:**
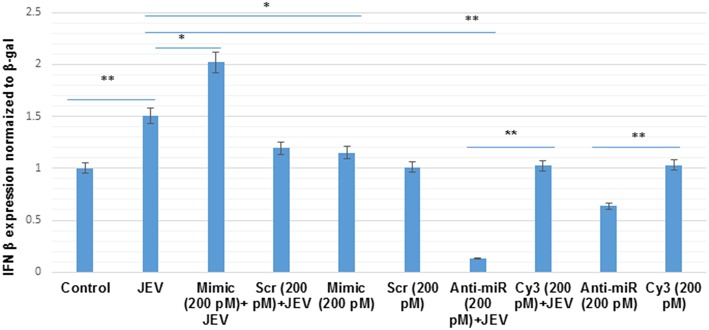
The hsa-miR-374b-5p activates type-I interferon in JEV infected human microglial cells. The graph shows the up-regulation of IFN-β level by 2-fold during microRNA over-expression and JEV infection while the anti-miR during JEV infection suppresses the IFN-β level by 90%. The scramble and cy3 anti-miR has been used as a control to further prove the specificity of microRNA, hsa-miR-374b-5p during JEV infection. In addition, the over-expression alone did increases the IFN-β level by 1.1-fold but it was comparatively lower than both the over-expression and JEV infection. The data are shown as mean ± S.E from three independent experiments (*n* = *3*). The fold change is significant where * denotes *P* < 0.05, ** denotes *P* < 0.01, *** denotes *P* < 0.001.

The phosphorylated form of AKT (p-AKT) activates IRF3 (Joung et al., 2011; Lu et al., 2011). The p-IRF3 in turn promote the type-I interferon response (Chang et al., 2006; Tarassishin et al., 2011a,b). To study the effect of hsa-miR-374b-5p on type-I interferon response during JEV infection, IFN-β luciferase plasmid were co-transfected with microRNA mimics, scramble, anti-miR and cy3 anti-miR negative control in human microglial cells for 48 h. After, 24 h of transfection, JEV infection at MOI 5 was given for 24 h. The result shows a 2-fold up-regulation of IFN-β response upon over-expression of the mimic (200 pM) during JEV infection, while the inhibition of hsa-miR-374b-5p using anti-miR, during JEV infection results in the 90% suppression. In addition, the type-I interferon response is mediated via hsa-miR-374b-5p, no JEV infection was given during over-expression and knock-down experiments (48 h). The IFN-β levels increase 1.1-fold during mimic (200 pM) conditions. Similarly, the inhibition (anti-miR 200 pM) experiments suppress 40% of IFN-β level which is less than the combination of JEV and inhibitor together (Figure 7).

## Discussion

The viral infection initiates an early innate anti-viral immune response by type-I interferon (IFN-α/β) during the early phases of infection (Samuel, 2001; Randall and Goodbourn, 2008). The viral sensors in the cells induce the anti-viral responses through different pathways, such as the RIG-I and TLR3 pathways (Jiang et al., 2014), resulting in the production of type-I interferon. We and others earlier reported the modulation of interferon response during JEV infection (Manocha et al., 2014; Sharma et al., 2015; Ye et al., 2017). The JEV have devised several strategies to subvert the innate immune response in order to establish in host (Lee et al., 2005; Sharma et al., 2016). The PI3K/AKT pathway has been reported to play important roles in generation of immune responses either by positively or negatively regulating the downstream factors like, GSK3β, AP-1, NF-κB, NF-AT, CREB and JAK-STAT (Sarkar et al., 2004; Hazeki et al., 2007; Polumuri et al., 2007; Radler et al., 2017). In addition, the involvement of the PI3K/AKT pathway has also been reported in the expression of type I and type III interferon responses (Nguyen et al., 2001; Rani et al., 2002; Cianciulli et al., 2016). The activation of PI3K/AKT pathway results in the production of interferon response via IRF3 transcription factor (Tarassishin et al., 2011b; Cianciulli et al., 2016; Tang et al., 2017; Yang et al., 2017).

The PTEN (Phosphatase and tensin homolog) is a dual phosphatase and a negative regulator for PI3K/AKT pathway. The PTEN protein is involved in myriad of cellular functions and its dysregulation has been reported in various types of cancers (Xiao et al., 2016; Khalid et al., 2017; Shen et al., 2019). However, many reports highlighted the aberrant expression of PTEN in neurodegenerative disorders, neuroinflammation, neuropathic pain, and microglial polarization (Ning et al., 2004; Choi et al., 2005; Griffin et al., 2005; Zhao et al., 2014; Wang et al., 2015; Cao et al., 2017). In addition, the microbes mediated-innate immune responses via PTEN have been recently reported (Li et al., 2016). The present study demonstrated the modulation of PTEN at different time points during JEV infection in human microglial cells. The dephosphorylated PTEN is an active form of PTEN, which suppressed the p-PTEN levels and led to the activation of PI3K/AKT pathway (Vazquez et al., 2001; Chen et al., 2012b; Zhao et al., 2014). The phosphorylation of AKT at ser473 led to the activation of interferon regulatory factor 3 (IRF3) by phosphorylating the IRF3 at ser396 (Tarassishin et al., 2011a,b). We earlier reported the generation of innate-antiviral immune response by a negative regulator, TRIM21 which resulted in the induction of type-I interferon via IRF3 activation (Manocha et al., 2014). A similar response has been shown by PTEN during JEV infection, where the suppression of PTEN resulted in the activation of AKT proteins. The activated AKT, (p-AKT) phosphorylated the IRF3 expression (p-IRF3) at early time points during JE infection. The PI3K/AKT signaling pathway has been reported to be exploited by Flaviviruses in promoting viral entry, replication and blocking the apoptotic pathway (Lee et al., 2005; Das et al., 2010; Chen et al., 2017) in order to establish inside the host. On the other hand, the robust production of type-I interferon responses through the activation of PI3K/AKT pathway has been reported at early time point during microbial infections (Nguyen et al., 2001; Rani et al., 2002; Hazeki et al., 2007). The same has been observed in our previous study, where we have shown the increased levels of type-I interferon expression at the early time points of 12 h and 24 h during JEV infection (Manocha et al., 2014). Hence, the JEV modulated the p-PTEN/PTEN at an early time point to enter and replicate, while at the later stages of the infection's progression, the virus suppressed the PTEN and shut down the PI3K/AKT/IRF3 axis to suppress the type-I interferon response.

MicroRNAs have been reported to be involved in the immune suppression during viral infections (Sharma et al., 2016; Rastogi et al., 2018). The hsa-miR-374b-5p has been extensively reviewed in various disorders, neurodegenerative disorders, encephalopathy, amyotrophic lateral sclerosis (ALS), and cardiovascular disorders (Bian et al., 2019). In this study we demonstrated the novel role of hsa-miR-374b-5p in immune activation during JEV infection. We demonstrated the up-regulation of microRNA, hsa-miR-374b-5p during JEV infection (24 h). Further, the target prediction databases (TargetScan, miRdb, and miRWalk) identified PTEN as one of the putative targets. We demonstrated the targeting of the PTEN by microRNA, hsa-miR-374b-5p during JEV infection (24 h) by utilizing over-expression and knock-down approaches. To further validate the involvement of AKT and IRF3 in type-I interferon activation, the expression levels of p-AKT/AKT and p-IRF3/IRF3 proteins were confirmed by immunoblotting. We observed the activation of the IFN-β promoter during over-expression and suppression studies by using microRNA mimics and inhibitors, respectively, during the JEV infection (Figure 7). In addition, to delineate the effects of over-expression and knock-down, the IFN-β levels were studied without JEV infection, where there was a comparatively lower expression of IFN-β levels. Our findings have been complemented by the previously published reports, where the p-AKT was reported to activate IRF3, which resulted in the activation of type-I interferon responses (Rani et al., 2002; Randall and Goodbourn, 2008; Tarassishin et al., 2011b). In summary, we reported the modulation of PI3K/AKT/IRF3 axis by the negative regulator, PTEN via microRNA hsa-miR-374b-5p mediated pathway during JEV infection. However, the single microRNA can target multiple genes and the multiples genes can be targeted by multiple miRNAs or single miRNA. So, this PI3K/AKT/IRF3 axis might also get modulated by other microRNAs either by targeting the same or other genes. Therefore, further studies are required to understand the involvement of other microRNAs in the modulation of PI3K/AKT/IRF3 axis during JEV infection. The activation of PI3K/AKT at early stages of the infection might be helpful for JEV internalization and the suppression at the later stages indicated the ability of JEV in modulating the host's cellular anti-viral response as a part of immune evasion during the course of infection.

## Data Availability

The datasets generated for this study are available on request to the corresponding author.

## Author Contributions

MR conducted all of the experiments and wrote the manuscript. SS conceived the concept and guided throughout the experiments and contributed to the manuscript preparation.

### Conflict of Interest Statement

The authors declare that the research was conducted in the absence of any commercial or financial relationships that could be construed as a potential conflict of interest.

## Abbreviations

BSA: bovine serum albumin
DENV: Dengue virus
DMEM: Dulbecco's modified eagle medium
IFN-β: interferon β
IRF3 and 7: interferon regulatory factor 3 and 7
ISGs: interferon stimulated genes
ISRE: IFN-stimulated response elements
JEV: Japanese Encephalitis Virus
Mda5: Melanoma differentiation-associated protein 5
MOI: multiplicity of infection
p-PTEN: phosphorylated phosphatase and tensin homolog
PS: porcine kidney cells
PTEN: phosphatase and tensin homolog
PVDF: polyvinylidene fluoride
RIGI: retinoic acid inducible gene-I
TBEV: Tick Borne Encephalitis Virus
TLR3 and 4: Toll-like receptor 3 and 4
TRIF: TIR-domain-containing adapter-inducing interferon-β
WNV: West Nile Virus
YFV: Yellow Fever Virus
ZIKV: ZIKA virus.
